# A review on mTOR inhibitor use and outcomes of COVID-19 among patients with kidney transplantation

**DOI:** 10.6026/973206300211447

**Published:** 2025-06-30

**Authors:** Sawai Singh Rathore, Vanessa Vidaurre Corrales, Ashwin Hassan Gopala, Hamam Aneis, Ibrahim Marouf Yasin Al Shyyab, Mutaz AlBeetar, Samah Mohamed Kannas, Omar Jihad Saleh Almistarihi, Mohamed Hamed Daoud

**Affiliations:** 1 Department of Internal Medicine, Dr. Sampurnanand Medical College, Jodhpur, Rajasthan, India; 2 Department of Internal Medicine, Universidad Privada del Valle, Cochabamba, Bolivia; 3 Department of Internal Medicine, Jinzhou Medical College, Jinzhou, China; 4 Department of Internal Medicine, UPMC, Mckeesport, Pennsylvania; 5 Department of Internal Medicine, University of Sharjah, Sharjah, UAE; 6 Department of Internal Medicine, Fakeeh University Hospital, Dubai, UAE; 7 Department of Internal Medicine, Bogomolets National Medical University, Kyiv, Ukraine

**Keywords:** Kidney transplant, COVID-19, SARS-CoV-2, renal transplant, hospitalization, ICU admission, mortality, mTOR inhibitors, irolimus, everolimus, temsirolimus

## Abstract

Kidney transplant recipients are at increased risk of severe COVID-19 due to immunosuppression and the impact of mTOR inhibitors on outcomes remains unclear. Hence, we evaluated 24 observational studies with 5,882 kidney transplant patients to assess the association of mTOR inhibitors with COVID-19 severity and mortality. Random-effects models showed that mTOR inhibitors were significantly associated with reduced mortality (OR=0.63, 95% CI 0.48-0.83, P=0.001) but not with COVID-19 severity (OR=0.70, 95% CI 0.41-1.20, P=0.865). Thus, mTOR inhibitors may provide a survival benefit in kidney transplant patients with COVID-19, highlighting the need for further research.

## Background:

Due to their weakened immune systems, patients face a heightened risk of severe illness and adverse outcomes from COVID-19 [1]. Understanding the impact of various immunosuppressive treatments on COVID-19 outcomes is crucial to enhancing patient care and guiding clinical decisions [1]. In this context, the use of mTOR (mammalian target of rapamycin) inhibitors in kidney transplant patients is of particular interest [2]. Due to their immuno-modulatory qualities and potential advantages in preventing graft rejection, mammalian targets of rapamycin inhibitors, such as sirolimus and everolimus, have attracted a lot of interest in the field of transplantation [3]. The mTOR pathway, which controls cellular development, proliferation and immune responses, is the target of these drugs [4]. In order to reduce the risk of rejection after kidney transplantation, mTOR inhibitors are frequently used. These medications have been shown to be effective in lowering the frequency of acute rejection episodes and enhancing long-term graft survival [5]. Nevertheless, research into and discussion about their effects on viral infections, such as COVID-19, is still ongoing. Optimizing patient care and informing clinical decision-making requires an understanding of how the use of mTOR inhibitors as immunosuppressive agents affects COVID-19-related outcomes in kidney transplant patients [6, 7]. The relationship between the use of mTOR inhibitors and COVID-19 outcomes in kidney transplant recipients has been studied in a growing body of literature [8]. However, there isn't much agreement on the overall impact of mTOR inhibitors in this particular patient population because individual studies frequently report conflicting results [9]. Therefore, it is of interest to review on mTOR inhibitor use and outcomes of COVID-19 among patients with kidney transplantation.

## Methods:

Preferred Reporting Items for Systematic Reviews and Meta-analyses (PRISMA) guidelines are adhered to for the current systematic review and meta-analysis [10].

## Search strategy:

A comprehensive literature search was conducted with PubMed, Google Scholar and Embase until December 6th, 2024. We combined Medical Subject Headings (MeSH) terms and keywords and subsequent search terms were, (([Coronavirus] or [Covid-19] or [SARS-CoV-2] AND [Kidney transplant] or [mTOR inhibitors] or [Immunosuppression] or [Outcomes])). Language restrictions were not imposed and investigations were included from around the globe. To identify additional qualifying studies, we manually reviewed the reference lists of the included studies and relevant literature. Duplicate citations were removed and the remaining articles were assessed for eligibility based on their titles and abstracts. The PRISMA flow diagram is depicted in Figure 1 (see PDF).

## Eligibility criteria:

All eligible studies were included in this meta-analysis. For inclusion in this meta-analysis, the article had to meet the following criteria: (a) an article describing COVID-19 infection in kidney transplant patients and the use of mTOR inhibitors as immunosuppression; (b) studies with a sample size of 5 patients or more. These studies were included regardless of the patient's age, gender, or ethnicity. The following exclusion requirements were established in advance: (a) studies with no data on COVID-19 patient outcomes; (b) duplicate publications; (c) letters to the editor, case reports, commentaries, reviews and posters. After applying these requirements, a thorough analysis of the remaining studies was conducted.

## Study selection and quality assessment:

Two authors reviewed the titles and abstracts of the previously found papers independently. They used predetermined eligibility criteria to determine which studies to include. In case of any disagreements, a third author would assess and help resolve the conflict through negotiation and prior agreement. We used the Newcastle-Ottawa Scale (NOS) to evaluate bias risk and assess the quality of the included studies [11]. Two of us independently used the NOS to assess the quality of each study. Each study was rated as having low bias risk (8-9 points), moderate bias risk (5-7 points), or high bias risk (0-4 points) in different sections.

## Data extraction:

Two authors independently conducted the data extraction for each study, which was then cross-checked to minimize errors. Several details were collected from each study, including the first author's name, year of publication, the country where the study was conducted, study design, number of kidney transplant patients infected with COVID-19, number of hospitalized patients, all-cause mortality, median age and gender distribution (proportion of females).

## Statistical analysis:

The statistical software utilized for all statistical analyses was MedCalc® Statistical Software version 19.6.4 (MedCalc Software Ltd, Ostend, Belgium; https://www.medcalc.org; 2021). The results for outcome analysis were presented as odds ratios (ORs) with 95% confidence intervals (CIs) and pooled using the Mantel-Haenszel random-effects model. The I2 statistic was used to assess the heterogeneity of effect size estimates across the studies, categorized as low heterogeneity (I2 ≤ 25%), moderate (25-50%), or high (> 75%). Publication bias was explored using funnel plots, Egger's regression test and Begg-Mazumdar's rank correlation test.

## Grading quality of evidence:

Using the GRADE (Grading of Recommendations Assessment, Development and Evaluation) working group technique, we rated the quality of the evidence for the primary and secondary outcomes as high, moderate, low and very low (Table 2 - see PDF) [12, 13].

## Results:

The initial search across multiple databases resulted in 2, 219 articles. After removing duplicate studies, 1,056 articles were evaluated. 914 articles were further eliminated based on title and abstract review, leaving 74 papers for detailed analysis. Ultimately, 24 articles, comprising 5,882 kidney transplant recipients, met the inclusion criteria for this meta-analysis [9, 14, 15, 16, 17, 18, 19, 20, 21, 22, 23, 24, 25, 26, 27, 28, 29, 30, 31, 32, 33, 34, 35-36]. The median age of the included patients was 57 years, with 36% being female. Table 1 (see PDF) provides a summary of the baseline characteristics, demographics and study details of the patients. A meta-analysis result showed that using mTOR inhibitors in kidney transplants does not significantly increase the likelihood of severe COVID-19 infection compared to other forms of immunosuppression. (OR= 0.70, 95% CI 0.41 to 1.20, P= 0.865). There was no significant heterogeneity (I2 = 0%). This conclusion was based on data from 12 studies involving 771 renal transplant patients with COVID-19 (Figure 2 - see PDF). The meta-analysis also found that using mTOR inhibitors in kidney transplant patients is linked to lower odds of death from COVID-19 compared to other forms of immunosuppression (OR= 0.63, 95% CI 0.48 to 0.83, P= 0.001). This result showed no heterogeneity (I2 = 0%) and was based on data from 17 studies involving 4729 renal transplant patients with COVID-19 (Figure 3 - see PDF). Leave one out of the sensitivity analysis for mortality analysis doesn't change the result by a significant amount. Removal of Requião-Moura et al. leads to similar outcomes with a new OR of 0.71 (95% CI 0.51 to 0.98, P= 0.001). A similar effect was seen for severity analysis, expressing the robustness of our result. The risk of bias and quality of the studies included were assessed using the Newcastle-Ottawa Scale (NOS). Out of 24 studies, 6 were rated as high quality and 18 were rated as moderate quality, with an average score of 7 (Table 1 - see PDF). Overall, the evidence used in the analyses was of moderate quality. The standard funnel plots for the severity and mortality analysis showed moderate symmetry (Figure 5A - see PDF & 5B - see PDF). We further used Egger's regression test and Begg-Mazumdar's rank correlation test to evaluate publication bias. A significance level of p < 0.05 was considered to indicate publication bias. There was no evidence of publication bias in the severity analysis (Egger's test, P = 0.636; Begg-Mazumdar's rank correlation test, P = 0.410) and mortality outcome analysis (Egger's test, P = 0.007; Begg-Mazumdar's rank correlation test, P = 0.161). Therefore, these analyses were concluded to be without publication bias.

## Discussion:

In this meta-analysis, the severity and mortality outcomes of COVID-19-infected kidney transplant patients were examined in relation to the use of mTOR inhibitors as immunosuppressive drugs. By combining data from various studies, we sought to provide a thorough understanding of the effects of mTOR inhibitors in this particular patient population. Regarding the severity outcome, our meta-analysis showed that the use of mTOR inhibitors in Kidney transplant patients has no significant association with the odds of severe COVID-19 infection compared to the other forms of immunosuppression (OR= 0.70, 95% CI 0.41 to 1.20, P= 0.865). The absence of heterogeneity (I2=0%) indicates a high level of agreement among the included studies, supporting the robustness of the findings. However, our meta-analysis revealed a significant association between the use of mTOR inhibitors and reduced odds of mortality from COVID-19 infection compared to other immunosuppressive agents (OR=0.63, 95% CI 0.48 to 0.83, P=0.001). This finding suggests a potential survival benefit for kidney transplant patients receiving mTOR inhibitors. The lack of heterogeneity (I2=0%) indicates consistent results across the included studies. One possible explanation for the protective effect of mTOR inhibitors in kidney transplant patients with COVID-19 is their immunomodulatory properties. mTOR inhibitors, such as sirolimus and everolimus, target the mechanistic target of the rapamycin (mTOR) pathway, which plays a crucial role in regulating immune cell activation, proliferation and differentiation [37]. These drugs can modulate the immune response by inhibiting mTOR, which may result in a more balanced and controlled immune response to viral infections [38]. In particular, it has been demonstrated that mTOR inhibitors prevent the proliferation and activation of T cells, which are vital for both the onset of excessive inflammation and the immune response to viruses [39].

MTOR inhibitors may prevent excessive immune activation by lowering T cell activation, which would lessen the cytokine storm and subsequent tissue damage that are frequently linked to severe COVID-19 [40]. Additionally, mTOR inhibitors have been reported to promote regulatory T cell (T reg) expansion, which can further modulate the immune response and potentially limit the development of severe disease [41]. The antiviral abilities of mTOR inhibitors have also been demonstrated against a variety of viruses, including respiratory viruses [42]. They can inhibit viral replication, reduce viral load and enhance the host's antiviral defense mechanisms. In the context of COVID-19, mTOR inhibitors may interfere with the replication and spread of the SARS-CoV-2 virus, leading to a milder infection and improved outcomes in kidney transplant patients [43]. Figure 4 (see PDF) depicts the mechanism of the protective effect of mTOR Inhibitors in Kidney transplant patient infected with COVID-19. Our findings are consistent with several previously published studies investigating the impact of mTOR inhibitors on viral infections. An article exploring the effects of mTOR inhibitors on respiratory viral infections in solid organ transplant recipients reported a lower risk of mortality in patients receiving mTOR inhibitors [44]. It is important to note that not all studies have reported a beneficial effect of mTOR inhibitors in the context of viral infections. Some studies have suggested that mTOR inhibitors may impair the innate immune response, potentially leading to an increased risk of viral replication and severe disease [45]. These conflicting findings highlight the complexity of the immune response and the need for further research to understand better the precise mechanisms underlying the effects of mTOR inhibitors in viral infections. It is important to acknowledge some limitations of this meta-analysis. Firstly, although we observed no significant heterogeneity, the included studies varied in design, patient characteristics and specific mTOR inhibitors used, which may introduce some degree of variability. Additionally, the majority of the included studies were retrospective in nature, which may be prone to bias. Therefore, caution should be exercised when interpreting these results and further prospective studies are warranted to validate our findings. Future research directions should focus on elucidating the underlying mechanisms by which mTOR inhibitors exert their protective effects in kidney transplant patients with COVID-19. Additionally, investigations into the optimal dosing and timing of mTOR inhibitors in this patient population may help optimize their benefits while minimizing potential risks.

## Conclusion:

Known data shows mTOR inhibitors may reduce mortality in kidney transplant patients with COVID-19 compared to other immune-suppressants. However, they do not significantly impact disease severity. Further studies are needed to validate these findings and overcome current study limitations.

## Disclosure:

Authors have no potential conflicts of interest to disclose.

## Data availability statement:

The data that support the findings of this study are available from the corresponding author upon reasonable request.

## Funding statement:

No funding sources to declare

## Ethical Statement:

As it is a systematic review and meta-analysis based on existing literature, ethical approval and informed consent from patients are not required.

## Abbreviations:

COVID‐19: Coronavirus disease-2019

SARS-CoV-2: Severe acute respiratory syndrome coronavirus 2

mTOR: mammalian target of rapamycin
